# Puberty in beef heifers: effects of prenatal and postnatal nutrition on the development of the neuroendocrine axis

**DOI:** 10.1590/1984-3143-AR2024-0048

**Published:** 2024-08-12

**Authors:** Sarah West, Viviana Garza, Rodolfo Cardoso

**Affiliations:** 1 Department of Animal Science, Texas A&M University, College Station, TX, USA

**Keywords:** heifers, hypothalamus, nutrition, puberty

## Abstract

Reproductive maturation is a complex physiological process controlled by the neuroendocrine system and is characterized by an increase in gonadotropin-releasing hormone (GnRH) and luteinizing hormone (LH) pulsatile secretion. Nutrition during early development is a key factor regulating puberty onset, which is defined as first ovulation in females. In heifers, nutrient restriction after weaning delays puberty, whereas elevated levels of nutrition and energy reserves advance reproductive maturation. Recent studies in cattle and other animal models have shown that the dam’s nutrition during gestation can also program the neuroendocrine system in the developing fetus and has the potential to alter timing of puberty in the offspring. Among the metabolic signals that modulate brain development and control timing of puberty is leptin, a hormone produced primarily by adipocytes that communicates energy status to the brain. Leptin acts within the arcuate nucleus of the hypothalamus to regulate GnRH secretion via an upstream network of neurons that includes neurons that express neuropeptide Y (NPY), an orexigenic peptide with inhibitory effects on GnRH secretion, and alpha melanocyte-stimulating hormone (αMSH), an anorexigenic peptide with excitatory effects on GnRH neurons. Another important population of neurons are KNDy neurons, neurons in the arcuate nucleus that co-express the neuropeptides kisspeptin, neurokinin B, and dynorphin and have strong stimulatory effects on GnRH secretion. Our studies in beef heifers indicate that increased nutrition between 4 to 8 months of age advances puberty by diminishing NPY inhibitory tone and by increasing excitatory inputs of αMSH and kisspeptin, which collectively lead to increased GnRH/LH pulsatility. Our ongoing studies indicate that different planes of nutrition during gestation can alter maternal leptin concentrations and promote changes in the fetal brain. Nonetheless, at least in *Bos indicus*-influenced heifers, deficits programmed prenatally can be overcome by adequate postnatal nutrition without negatively impacting age at puberty or subsequent fertility.

## Introduction

Reproductive maturation is an intricate physiological process that involves physical and behavioral modifications rising from the activation of the hypothalamic-pituitary-ovarian (HPO) axis and subsequent establishment of estrous cycles in females (Sisk and Foster, 2004). Puberty involves a gradual activation of gonadotropin-releasing hormone (GnRH) neurons and an associated rise in GnRH and luteinizing hormone (LH) pulsatile secretion which ultimately support the final stages of follicular development and first ovulation (Ryan and Foster, 1980). Several physiological and environmental factors can impact the timing of puberty attainment. Among those factors, nutrition is likely one of the most important, having significant implications to human health and animal production. Studies in humans and multiple animal models, including rodents, sheep, and cattle clearly indicate that increased nutritional levels during prepubertal development advance onset of puberty in females (Ryan and Foster, 1980; Amstalden et al., 2011).

Age at puberty is one of the most important traits dictating lifetime productivity in replacement beef heifers. It is expected that most heifers in beef production will attain puberty around 12 to 14 months of age in order to conceive early during their first breeding season, and to have their first calf by approximately 2 years of age (Lesmeister et al., 1973; Cardoso et al., 2020). However, a large proportion of beef heifers in the U.S. and globally fail to reach these production goals (Hughes, 2013). This is particularly relevant for later-maturing heifers, such as *Bos indicus*-influenced and large frame heifers, in which the skeletal size required to support a healthy and safe pregnancy is often attained well before puberty. Therefore, several managerial strategies, including nutritional approaches, have been developed to program the neuroendocrine axis of these later-maturing heifers to successfully induce early puberty and meet the production goals mentioned above. One area that remains less clear and requires additional studies is the potential impact of maternal nutrition during gestation on the development of the offspring’s neuroendocrine system and subsequent reproductive maturation. Our most recent studies and those of others have started to shed some light on the effects of maternal nutrition on brain development and puberty attainment in the heifer offspring. In this review, we present a brief overview of the neuroendocrine mechanisms controlling reproductive maturation in heifers, summarize the effects of prenatal and postnatal nutrition on this process, and discuss some nutritional approaches that are currently available to advance puberty in replacement beef heifers. For more in-depth discussion of the hypothalamic mechanisms controlling puberty in female ruminants and effects of nutrition on puberty in cattle and sheep, the reader is referred to previous reviews (Amstalden et al., 2011; Garza et al., 2023).

## Neuroendocrine mechanisms and metabolic regulation of puberty

The hypothalamus is the primary component of the HPO axis controlling timing of puberty in female mammals (Ryan and Foster, 1980; Sisk and Foster, 2004; Cardoso et al., 2018). In heifers, approximately 60 days prior to first ovulation, the low-frequency pulsatile secretion of GnRH into the hypothalamic-pituitary portal vasculature that characterizes the prepubertal stage ultimately transitions into a higher-frequency mode of secretion, which initiates the peripubertal transition period (Day et al., 1987). The increase in hypothalamic GnRH secretion is accompanied by an increase in the frequency of corresponding LH pulses by gonadotrophs in the anterior pituitary. The increased LH pulsatile release supports final maturation of antral follicles, which in turn produce higher amounts of estradiol to activate the estradiol positive feedback mechanism and induce first ovulation (Foster and Ryan, 1979; Day et al., 1984).

The pulsatile secretion of LH in the peripheral circulation is directly associated with the synchronous activation of hypothalamic GnRH neurons (Clarke and Cummins, 1982). The process of concomitant and synchronous activation of multiple GnRH neurons resulting in a GnRH pulse release into the hypothalamic-pituitary portal vasculature is referred to as the GnRH pulse generator (Knobil, 1980; Goodman and Karsch, 1981). Recent studies have started to elucidate the cellular and molecular mechanisms within the hypothalamus that control this synchronous activation of GnRH neurons required for pulse generation (Cheng et al., 2010; Lehman et al., 2010b; Redmond et al., 2011; Grachev et al., 2016). In most mammals, including cattle and sheep, a subset of neurons within the arcuate nucleus of the hypothalamus co-expresses three neuropeptides: kisspeptin, neurokinin B (NKB) and dynorphin (Lehman et al., 2010a). These neurons, termed KNDy neurons (K = kisspeptin; N = NKB; and Dy = dynorphin), also contain receptors for the peptides NKB and dynorphin (Lehman et al., 2010b); but do not express receptors for kisspeptin (Lehman et al., 2010a). It has been postulated that the process of GnRH pulse generation occurs as KNDy neurons secrete kisspeptin in response to their autocrine and paracrine stimulation by NKB, which in turn leads to kisspeptin stimulation of GnRH neurons (Lehman et al., 2010a; Goodman et al., 2013; Goodman et al., 2014; Goodman et al., 2018). The activation of KNDy neurons promotes the secretion of dynorphin, which is an inhibitory neuropeptide that suppresses KNDy neuron activity. The sequential release of these three KNDy peptides is believed to control the synchronous activation of GnRH neurons, thus controlling GnRH pulse generation (Herbison, 2018). Importantly, the large majority of KNDy neurons contain estrogen receptor-α (ER-α or ESR1; Franceschini et al., 2006), suggesting that KNDy neurons comprise a key neuronal network by which estrogen can control GnRH/LH pulse secretion and puberty.

In prepubertal heifers, small concentrations of estradiol produced by ovarian follicles markedly inhibit LH secretion to a low frequency (1 pulse every 4-6 hours) due to an increase hypothalamic responsiveness to the estradiol negative feedback loop (Foster and Ryan, 1979; Day et al., 1987; Kinder et al., 1995). However, during the peripubertal transition (50-60 days prior to first ovulation), the sensitivity of the hypothalamus to the estradiol negative feedback gradually decreases, resulting in increased LH pulsatile secretion that supports development of preovulatory follicles (Day et al., 1984; Day et al., 1987). The premise of a gradual “escape” of the neuroendocrine system from the estradiol negative feedback is supported by the fact that estradiol’s inhibitory effects suppressing LH pulse frequency gradually decreases with age in ovariectomized heifers (Schillo et al., 1982). While there is strong evidence in cattle and sheep that KNDy neurons are involved in this process, the precise cellular and molecular mechanisms mediating the peripubertal reduction in the hypothalamic sensitivity to the estradiol negative feedback remain largely unknown.

**Figure 1:**
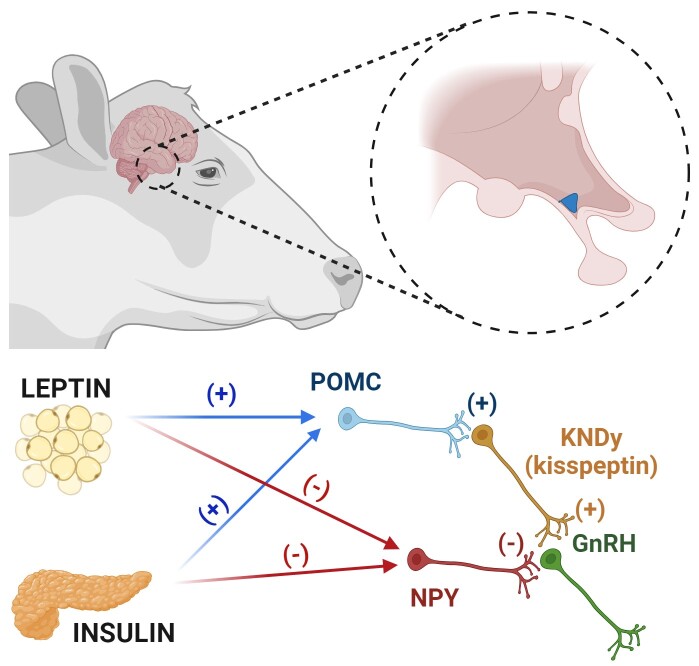
Nutritional and metabolic regulation of reproductive neuroendocrine function in heifers. The metabolic hormones leptin (produced primarily by adipocytes) and insulin (produced by the pancreas) act within the arcuate nucleus of the hypothalamus to modulate GnRH secretion. Since GnRH neurons do not express the receptors for these metabolic hormones, leptin and insulin must act through an upstream neuronal network. Neurons expressing proopiomelanocortin (POMC) are up-regulated by both leptin and insulin, resulting in greater synthesis and secretion of alpha melanocyte-stimulating hormone (α-MSH), which in turn stimulates kisspeptin secretion by KNDy neurons. Kisspeptin is a potent stimulator of GnRH release. Moreover, leptin and insulin suppress the expression of neuropeptide Y (NPY) within the arcuate nucleus, which has direct inhibitory effects on GnRH neurons. Therefore, the upregulation of POMC and downregulation of NPY collectively result in greater stimulation of GnRH neurons and subsequently greater GnRH/LH pulse frequency, which is a key physiological process driving puberty attainment in heifers. Created with BioRender.

Insulin, leptin, ghrelin, and insulin-like growth factor-1 (IGF1) are metabolic hormones that signal the metabolic status to the central nervous system (CNS) and regulate timing of puberty in heifers (Williams et al., 2002; Williams et al., 2018). While leptin, insulin, and IGF1 are elevated in conditions of higher nutritional planes and have stimulatory effects on GnRH/LH secretion (Williams et al., 2002; Williams et al., 2018), ghrelin, a hormone produced primarily by the stomach, is produced in greater amounts during periods of dietary restriction and suppresses GnRH/LH pulsatile secretion (Fernandez-Fernandez et al., 2005). Elevated rates of body weight gain (0.8 to 1.5 kg/d) during prepubertal development result in increased circulating concentrations of leptin, insulin and IGF1 in beef heifers (Garcia et al., 2002; Allen et al., 2017). Evidence from sheep and other animal models, such as transgenic mouse models, strongly suggest that among the metabolic signals, leptin is likely the main player signaling energy reserves to the hypothalamus and subsequently modulating GnRH secretion and timing of puberty. Leptin is a peptide hormone secreted primarily by adipocytes (Zhang et al., 2005) and its circulating concentrations are positively correlated with the amount of stored adipose tissue (Garcia et al., 2002; Allen et al., 2017). A peripubertal rise in leptin concentrations is a required, permissive signal for puberty attainment in heifers (Williams et al., 2002; Cardoso et al., 2018; Zieba et al., 2020). Moreover, substantial reductions in adiposity, such as those seen after chronic starvation, result in disruptions in estrous cyclicity, which continue until minimal levels of adipose reserves are restored (Frisch, 1985). In heifers and mature cows, leptin’s stimulatory effects on GnRH/LH secretion are limited primarily to periods of nutrient restriction (Amstalden et al., 2000; Amstalden et al., 2002; Amstalden et al., 2003), and these effects occur due to leptin’s direct action at both the hypothalamic and anterior pituitary levels (Amstalden et al., 2003; Zieba et al., 2004). The actions of leptin promoting GnRH and LH secretion during nutrient restriction may occur due to a decrease in the hypothalamic sensitivity to the estradiol negative feedback (Foster and Ryan, 1979; Ryan and Foster, 1980; Day et al., 1987; Kinder et al., 1987). Because GnRH neurons do not express the leptin receptor (Quennell et al., 2009) or ER-α (Dorling et al., 2003), an upstream neuronal network must convene the effects of leptin on GnRH release (Figure 1).

Metabolic hormones such as leptin, insulin, and IGF1 control appetite, energy expenditure, and reproduction at the CNS level by modulating the secretion of inhibitory and/or excitatory neuropeptides (Evans and Anderson, 2017). These metabolic signals regulate GnRH secretion primarily by acting at the arcuate nucleus, a hypothalamic region that abundantly expresses receptors for metabolic hormones (Cone et al., 2001). Among the different hypothalamic neuropeptides, neurons secreting proopiomelanocortin (POMC) and agouti-related peptide (AgRP)/ neuropeptide Y (NPY) are two critical mediators of leptin’s actions on GnRH neurons (Hill et al., 2008). POMC regulates appetite and energy balance via several systemic mechanisms and neuronal pathways (Candler et al., 2019). Increased feed intake and positive energy balance increase *POMC* expression, while nutrient restriction and negative energy balance reduce *POMC* expression within the arcuate nucleus (Caron et al., 2018). In regards the control of GnRH secretion and reproduction, POMC has stimulatory effects on the secretion of GnRH/LH during positive energy balance primarily by acting on KNDy neurons stimulating kisspeptin secretion. The effects of nutrition on *POMC* expression are primarily mediated via leptin since leptin stimulates *POMC* expression and release in the arcuate nucleus. These stimulatory effects of POMC neurons on kisspeptin secretion are likely mediated by the neuropeptide melanocyte-stimulating hormone α (αMSH), a product of the *POMC* gene (Seeley et al., 2004). With virtually opposing effects as those reported for POMC, NPY is an orexigenic peptide found in the arcuate nucleus (Guzman et al., 2019) that its expression is increased during feed restriction and negative energy balance. All NPY neurons express leptin receptors and leptin suppresses *NPY* expression and secretion within the arcuate nucleus. However, different from POMC neurons that act primarily via KNDy neurons, NPY neurons controls GnRH/LH secretion mainly via its direct inhibitory effects of GnRH neurons (Figure 1).

## Nutritional regulation of puberty: prenatal effects

Maternal nutritional extremes (undernutrition and overnutrition) during gestation may lead to physiological adaptations in the developing fetus, which in turn can alter the phenotype of the offspring through epigenetic modifications (Wang et al., 2012). During the second trimester of gestation in cattle, fetal organs continue to develop, along with muscle tissue, adipogenesis, and limb growth. The second and third trimesters of gestation have been reported to be a critical developmental window for programming the offspring’s phenotype in cattle. Particularly, the final trimester of gestation is the period in which 75% of fetal growth occurs (Eley et al., 1978). The effects of fetal programming have been studied in several species; however, the programming effects of maternal nutrition on reproductive function in cattle remain largely unknown. Moreover, studies investigating the potential effects of fetal programming in cattle involve a variety of models, including environmental stress, transportation stress, maternal breed (*Bos taurus* vs. *Bos indicus*), and dietary treatments (underfed vs. overfed). Therefore, those aspects must be taken into consideration since the phenotype programmed will likely be influenced by the type of prenatal insult, the magnitude of the insult, the period in which it occurs, as well as the type of animals investigated.

Adequate maternal nutrition during gestation is required for the proper fetal development; thus, several studies have been carried out to investigate the effects of gestational nutrition on the offspring’s reproductive function in cattle. Earlier studies reported that moderate nutrient restriction (60% of NRC requirements) during the first trimester of gestation had no effect on birth weight or follicle diameter in the heifer offspring using crossbred cattle (Mossa et al., 2013). However, prenatal nutrient restriction resulted in approximately a 50% reduction in antral follicle numbers compared to control heifers (Mossa et al., 2013). Notably, antral follicle count (AFC) is a predictor of the number of healthy follicles in a cow's ovarian reserve and likely an important marker for life-long fertility (Ireland et al., 2008). In line with the AFC data, heifers subjected to prenatal nutrient restriction had lower circulating concentrations of anti-mullerian hormone (AMH) and FSH compared to control heifers. Despite these changes in AFC and AMH concentrations, nutrient restriction during the first trimester of gestation did not have an impact on age at puberty (Ireland et al., 2008; Mossa et al., 2013). Despite these reported effects on ovarian reserves, more recent studies have reported that adequate nutrition later in gestation after nutritional restriction negates the adverse effects on growth of the fetus with little consequences on reproductive performance later in life (Bell and Greenwood, 2016; Noya et al., 2019).

Studies involving *Bos taurus* cows fed either a low (75% of NRC) or moderate (100% of NRC) diet during the second trimester of gestation followed by either a high (125% of NRC), moderate, or low diet during the third trimester of gestation were carried out by Cushman et al. (2014). These nutritional treatments did not affect age at puberty nor AFC; however, heifers born to dams on a high maternal diet calved 21 days earlier on average compared to heifers in the other nutritional groups (Cushman et al., 2014). Another study in crossbred cattle reported that maternal overnutrition during the second and third trimesters of gestation reduced the number of ovarian primordial follicles in female fetuses, suggestive of a decrease in the ovarian reserve and potential long-term fertility effects (Weller et al., 2016). However, this study was not carried out for a long-term to assess if the deleterious effects of prenatal nutrition on the offspring’s ovarian reserve were associated with altered ovarian physiology and overall fertility after reproductive maturation (Weller et al., 2016).

While the studies discussed above focused on important physiological markers for fertility, research investigating the effects of maternal nutrition during gestation on the neuroendocrine system of cattle remains scarce. To examine the effects of prenatal and early postnatal nutrition on neuroendocrine function, puberty, and subsequent fertility in *Bos indicus*-influenced heifers, a 3x2 factorial research model was developed by our research group (Maia et al., 2022). During the second trimester (6 months) of gestation, Brahman-influenced dams pregnant with a female fetus were fed to achieve body condition scores (BCS) of 3.5-4 (thin; L), 5.5-6 (moderate; M), or 7.5-8 (obese; H). Heifer offspring born to the three nutritional groups were early weaned at approximately 3.5 months of age and randomly assigned to a low gain (0.5 kg/d; L) or a high gain (1 kg/d; H) dietary treatment until ~8 months of age. This 3x2 factorial design resulted in 6 nutritional treatments: (LL, LH, ML, MH, HL, HH; Table 1). Dams BCS and body weight (BW) were assessed every 2 weeks and diets adjusted accordingly. At week 6 of the experiment, BCS and BW started to significantly differ among the treatment groups, and at parturition, BCS was 7.6 ± 0.12, 5.1 ± 0.12, and 3.3 ± 0.12 for H>M>L, respectively (Maia et al., 2022). Heifers born to nutrient-restricted cows had approximately a 10% reduction in birth weight compared to heifers born to obese dams but did not differ when compared to the moderate (control) group (Maia et al., 2022). Remarkably, neither age nor body weight at puberty were affected by the prenatal treatments, indicating that maternal nutrition during the last two trimesters of gestation does not delay or hasten puberty attainment of the heifer offspring in *Bos indicus*-influenced cattle (Table 1). Similarly, prenatal nutritional treatments did not affect preovulatory follicle size, dominant follicle growth rate, AFC, or endometrial thickness in the heifer offspring. Likewise, no treatment effects were observed for progesterone concentrations during the luteal phase or estradiol concentrations during the follicular phase after onset of puberty (Maia et al., 2022). At the tissue-level, prenatal dietary treatments had no significant effects on the total number of GnRH neurons, as well as the number of NPY- and kisspeptin-expressing neurons within the arcuate nucleus (West & Cardoso, unpublished observations).

**Table 1:** Interactive effects of prenatal and early postnatal nutrition on puberty attainment and subsequent fertility in *Bos indicus*-influenced heifers. Cows were fed to achieve a low (L; BCS: 3-3.5), moderate (M; BCS: 5.5-6), or a high body condition score (H; BCS: 7.5-8) between the second trimester and the end of gestation. Heifer offspring were weaned at 3.5 mo of age and fed either a low-gain (L) diet targeting a rate of gain of 0.5 kg/d or a high-gain (H) diet targeting a rate of gain of 1 kg/d from 4 to 8 months of age. This 3x2 factorial design resulted in 6 nutritional treatments: (LL, LH, ML, MH, HL, HH.

**Maternal** **Nutrition**	**Postnatal** **Nutrition**	**Maternal x Postnatal**	**Reproductive** **Phenotype**
Low (BCS 3-3.5)	Low-Gain (0.5 kg/day)	LL	· Delayed puberty (~15 mo of age)
			· Normal fertility during the first breeding season
	High-Gain (1 kg/day)	LH	· Early puberty (~12 mo of age)
			· Normal fertility during the first breeding season
Moderate (BCS 5.5-6)	Low-Gain (0.5 kg/day)	ML	· Delayed puberty (~15 mo of age)
			· Normal fertility during the first breeding season
	High-Gain (1 kg/day)	MH	· Early puberty (~12 mo of age)
			· Normal fertility during the first breeding season
High (BCS 7.5-8)	Low-Gain (0.5 kg/day)	HL	· Delayed puberty (~15 mo of age)
			· Normal fertility during the first breeding season
	High-Gain (1 kg/day)	HH	· Early puberty (~12 mo of age)
			· Normal fertility during the first breeding season

A comprehensive interpretation of previous research involving nutritional programming in cattle is challenging due to the many variables involved in those studies, namely the period of gestation, magnitude of restriction, *Bos indicus* vs. *Bos taurus*, cow vs. heifer, nutrition during postnatal life, and other stress factors that may have occurred during gestation. Based on our extensive studies using *Bos indicus*-influenced heifers, we postulate that maternal nutrition during the last two trimesters of gestation has only minimal, if any effects on age at puberty and subsequent fertility of the heifer offspring. We also propose that *Bos indicus*-influenced heifers might have an increased ability to adapt to extreme nutritional conditions during gestation, thus protecting their offspring to adverse developmental programming, since similar experimental paradigms result in more clear phenotypic alterations in *Bos taurus* cattle.

## Nutritional regulation of puberty: postnatal effects

Nutritional strategies that promote higher rates of body weight gain (1 kg/d) after weaning result in elevated circulating concentrations of key metabolic hormones, such as leptin, insulin, and IGF1, compared to diets that promote lower rates of body weight gain, such as 0.5 kg/d (Allen et al., 2012). These hormonal changes during early postweaning development result in greater pulsatile secretion of GnRH and LH and subsequently in an advancement in puberty attainment (Cardoso et al., 2014b). At the hypothalamic level, our studies demonstrated that higher rates of body weight gain resulted in a smaller percentage of GnRH neurons receiving inputs from NPY fibers (inhibitory) and fewer NPY-expressing neurons within the arcuate nucleus compared to heifers gaining body weight at lower rates (Alves et al., 2015; Figure 1). Because NPY has a well-defined inhibitory effect on GnRH secretion in cattle, we postulate that a decrease in *NPY* mRNA expression as well as a decrease in NPY projections towards GnRH neurons contribute to the increase in GnRH secretion. Moreover, an increased plane of nutrition postweaning resulted in higher numbers of *POMC*-expressing neurons within the arcuate nucleus and a greater number of αMSH projections (excitatory) to KNDy neurons (Cardoso et al., 2015). Because POMC, via αMSH, stimulates GnRH/LH secretion in female ruminants, we propose that the POMC-kisspeptin pathway is also an important mechanism by which increased planes of nutrition can stimulate GnRH secretion. Therefore, it appears that increased plane of nutrition during early postweaning development advances puberty in heifers by modulating both pathways the NPY-GnRH (inhibitory) and the POMC-kisspeptin (stimulatory) in an antagonistic manner (Figure 1). Taken together with our prenatal nutrition studies, our overall conclusion is that nutrition during the early postweaning period has a markedly greater impact on the neuroendocrine system of beef heifers compared to prenatal nutrition. While prenatal nutritional challenges (e.g., severe maternal undernutrition) likely promote modifications within the developing neuroendocrine axis, adequate nutrition postnatally can likely override these prenatally programmed deficits (Gorski et al., 2006).

In regards the developmental window in which the neuroendocrine system is more susceptible to the nutritional programming of early puberty, our previous research suggests that a critical window exists between 4 and 8 months of age in heifers (Cardoso et al., 2018). We have developed a stair-step compensatory growth program in which heifers were assigned to be fed 1 of 4 diets beginning at 4 months of age: 1) Low Control (LC), *Bos indicus*-influenced heifers had restricted feed intake of a forage-based diet to gain 0.5 kg/d until 14 months of age; 2) High Control (HC), heifers had limited feed intake of a high-concentrate diet to gain 1 kg/d until 14 months of age; 3) Stair-Step 1 (SS-1), heifers had *ad libitum* access to a high-concentrate diet until 6.5 months of age followed by restricted access to a high-forage diet to gain 0.35 kg/d until 9 mo of age, followed by *ad libitum* feed intake of a high-concentrate diet until 11.5 mo of age, and restricted feed intake of a high-forage diet to gain 0.35 kg/d until 14 mo of age; and 4) Stair- step 2 (SS-2), heifers were fed at a reverse sequence of SS-1, beginning with restricted access to a high-forage diet. As anticipated, heifers fed to gain body weight at a high rate (HC) attained puberty considerably earlier than heifers gaining only 0.5 kg/d (LC), with all HC heifers reaching puberty by 14 mo of age compared to only 40% of LC heifers. Of significance, heifers fed at a high rate of gain between 4 and 6.5 months of age (SS-1) achieved early puberty like the heifers fed the continuous high-gain diet (HC) throughout the entire study, despite a period of moderate nutrient restriction between 6.5 and 9 months of age. These findings indicate that neuroendocrine changes programmed between 4 and 6.5 months of age persist until puberty and are likely permanent modifications that may have life-long effects on reproductive function (Cardoso et al., 2014a). These results support the notion that brain plasticity progressively decreases between birth and puberty and that heifers are more sensitive to the effects of nutrition in programming early puberty during early postweaning development.

While the impact of postweaning nutrition on pubertal attainment has been extensively characterized in *Bos taurus* and *Bos indicus*-influenced beef heifers, the effects of preweaning nutrition remain less clear. One management strategy extensively used to manipulate preweaning nutrition and increase rate of body weight gain is the use of creep feeding. Energy and protein intake of calves is typically limited in extensive rangeland management cow-calf systems during summer and winter months, which negatively impacts pre- and post-weaning growth rates and has been shown to be associated with a delayed puberty in heifers (Guggeri et al., 2014). Using *Bos taurus* heifers, Buskirk et al. (1996) reported that the percentage of heifers attaining puberty linearly increased by approximately 30% as duration of creep feed supplementation increased, concluding that creep feed *ad libitum* access for up to 85 days prior to weaning accelerated puberty by increasing weight gain in heifers. However, in a different experiment using *Bos indicus* cattle, creep feeding Nellore heifers 110 to 205 days prior to weaning failed to significantly advance attainment of puberty (Nepomuceno et al., 2017). Likewise, Reis et al. (2015)provided *ad libitum*access to creep feeding from 68 to 118 days of age to nursing beef heifers and reported that age at puberty was also not impacted by creep feeding supplementation. In this study, creep feeding supplementation did not alter body composition nor increased circulating concentrations of metabolic hormones such as IGF1, leading the authors to propose that a longer period of creep feeding supplementation is likely needed to significantly alter the metabolic status of heifers and hasten puberty (Reis et al., 2015). In conjunction with our previous results using the stair-step nutritional model, we propose that *ad libitum* access to creep feeding for a longer period that overlaps with the critical window for programming the neuroendocrine axis (4 to 8 months of age) is likely needed to induce early reproductive maturation in beef heifers.

## Conclusions

In summary, studies performed by our research group and others demonstrate that early postweaning nutrition, particularly within the 4 to 8 months of age window, can program the developing neuroendocrine axis and markedly advance puberty in *Bos taurus* as well as *Bos indicus*-influenced heifers. These effects are largely mediated by leptin and insulin signaling within the arcuate nucleus of the hypothalamus and suppressing the NPY-GnRH inhibitory pathway and activating the POMC-kisspeptin excitatory pathway. At present, nutritional approaches such as the stair-step nutritional management, early weaning, and creep feeding are available to optimally time puberty in replacement beef heifers. Finally, while maternal nutrition during early gestation can alter ovarian development and function in *Bos taurus* cattle, maternal nutrient restriction during late gestation does not have significant effects on age at puberty and subsequent fertility of *Bos indicus*-influenced heifers.
